# Mysterious meiotic behavior of autopolyploid and allopolyploid maize

**DOI:** 10.3897/CompCytogen.v12i2.24907

**Published:** 2018-07-20

**Authors:** Muhammad Zafar Iqbal, Mingjun Cheng, Yanli Zhao, Xiaodong Wen, Ping Zhang, Lei Zhang, Asif Ali, Tingzhao Rong, Qi Lin Tang

**Affiliations:** 1 Sichuan Agricultural University, Maize Research Institute, Wenjiang 611130, Sichuan, China; 2 Sichuan Provincial Grassland Work Station, Chengdu 610041, China

**Keywords:** Maize, polyploidy, meiosis, GISH, chromosome stability, genome evolution

## Abstract

This study was aimed to investigate the stability of chromosomes during meiosis in autopolyploid and allopolyploid maize, as well as to determine an association of chromosomes between maize (Zea mays ssp. mays Linnaeus, 1753) and *Z. perennis* (Hitchcock, 1922) Reeves & Mangelsdor, 1942, by producing a series of autopolyploid and allopolyploid maize hybrids. The intra-genomic and inter-genomic meiotic pairings in these polyploids were quantified and compared using dual-color genomic in-situ hybridization. The results demonstrated higher level of chromosome stability in allopolyploid maize during meiosis as compared to autopolyploid maize. In addition, the meiotic behavior of *Z. perennis* was relatively more stable as compared to the allopolyploid maize. Moreover, ten chromosomes of "A” subgenome in maize were homologous to twenty chromosomes of *Z. perennis* genome with a higher pairing frequency and little evolutionary differentiation. At the same time, little evolutionary differentiation has been shown by chromosomes of "A” subgenome in maize, while chromosomes of "B” subgenome, had a lower pairing frequency and higher evolutionary differentiation. Furthermore, 5I^M^ + 5II^PP^ + 5III^MPP^ and 5II^MM^ + 5II^PP^ + 5IV^MMPP^ were observed in allotriploids and allotetraploids respectively, whereas homoeologous chromosomes were found between the "A” and "B” genome of maize and *Z. perennis*.

## Introduction

*Zea* Linnaeus, 1753, belongs to the tribe Maydeae Candolle, 1882, and consists of two sections: section Luxuriante and section Zea (Iltis and Doebley 1980, Wang et al. 2011). The domesticated maize (Zea mays ssp. mays Linnaeus, 1753, 2n=20, is also called corn, (belongs to genus *Zea* and is classified in section Zea) is an important economic crop and polyploid genetic model with many duplicated genes in all of its ten chromosomes (Ahn et al. 1993; Gaut et al. 2000; Wang et al. 2006; Wang et al. 2011). There are three models to explain these duplicated genes, which are multiple independent duplications occurring in one genome, autotetraploidy and allotetraploidy (Gaut and Doebley 1997, Molina et al. 2013). Besides maize, the other species of genus *Zea* are called teosinte, which provide an excellent system to study ecological genomics, population genetics and plant breeding (Hufford et al. 2012). *Z. perennis* (Hitchcock, 1922) Reeves & Mangelsdor, 1942 (section Luxuriantes), 2n=40 is one of the perennial teosintes having an inferred octoploid origin (González et al. 2006, Wang et al. 2011). In a previous study, *Z. perennis* was thought to have originated from a *Z. diploperennis* Iltis, Doebley & Guzman, 1979 - like ancestor (Tiffin and Gaut 2001).

From previous research, it is evident that crosses could be made between maize and *Z. perennis*; as a consequence the genomic relationship was assessed by meiotic pairing analysis of hybrids between both species (Longley 1924, Emerson and Beadle 1930, Mangelsdorf 1939, Shaver 1964, Poggio et al. 1999, Cao et al. 2002, TANG et al. 2004, Gonzalez and Poggio 2011). Different meiotic pairings were reported in their allopolyploid hybrids; the allotriploid, synthesized from a cross between diploid maize and *Z. perennis*, and the allotetraploid was developed by a cross between tetraploid maize and *Z. perennis*. Moreover, less meiotic stability and fertility has been observed in allotriploids as compared to allotetraploids (Tang et al. 2005). The common meiotic configurations of two allopolyploids were 5I+5II+5III in allotriploid hybrids and 10II+5IV in allotetraploid hybrids (Longley 1924; Molina and Garcia 1999; González et al. 2006). Maize and *Z. perennis* have basic chromosome number x = 5, and hypothetical formulas for maize and *Z. perennis* are "A_m_A_m_B_m_B_m_” and "A_p_A_p_A_p’_A_p’_B_p1_B_p1_B_p2_B_p2_”, respectively (Naranjo et al. 1994). Besides, the existence of a controversy about the origin of "A” and "B” subgenomes, there might be two possible mechanisms behind the evolution, one mechanism refers to a duplication event that might have occurred in the "A” genome of a diploid species followed by evolutionary differentiation that converted "A” genome into homoeologous sub genomes "A” and "B”; the other proposes that the genome composition of "AABB” hybrids might be the result of an ancestral cross between two closely related "AA” and "BB” genomes followed by evolutionary fractionation (Molina et al. 2013). Furthermore, previous studies demonstrated that "A” subgenome in maize and *Z. perennis* showed higher homology of chromosomes, as well as, suffered fewer gene losses and higher level of gene expression as compared to the "B” subgenome, while "B” subgenome had a faster differentiation that led to species isolation and eventually resulted in the formation of different species of *Zea* (Freeling and Thomas 2006, Swanson-Wagner et al. 2010, Schnable et al. 2011). However, both hypotheses could not explain differences within "A” and "B” subgenomes of both maize and *Z. perennis* clearly. In all, there is limited understanding about relationship of chromosomes between maize and *Z. perennis*, therefore chromosome stability of both autopolyploid and allopolyploid maize was investigated in current study with the following objectives: (i) to give systematic understanding of chromosome relationship between maize and *Zea perennis*; (ii) to observe the meiotic chromosome stability in autopolyploid and allopolyploid maize (iii) to reveal the origin and differentiation process of "A” and "B” subgenomes (iv) to validate the chromosome paring pattern by using general cytology and dual-color genomic in situ hybridization (GISH) in a number of autopolyploid and allopolyploid hybrids that were synthesized by the cross of maize and *Zea perennis*.

## Material and methods

### Abbreviations

**GISH** Genomic *in situ* Hybridization

**RCC** Relative chaotic coefficient

**PMCs** Pollen mother cells

### Plant material

Plant materials are shown in Table 6. Maize inbred line wf9 (2n=2x=20) and a tetraploid maize Twf9 (2n=4x=40) (derived from chromosome doubling of wf9) were provided by the United States Department of Agriculture (USDA), *Zea perennis* (2n=4x=40, accession no. 9475) was obtained from International Maize and Wheat Improvement Center (CIMMYT). The plant material was raised at experimental farm of Sichuan Agricultural University, Jinghong, China. Three crosses were made by hand pollination that were (1) between diploid maize inbred wf9 and *Z. perennis*, (2) between tetraploid maize Twf9 and *Z. perennis* and (3) between diploid maize inbred line wf9 and tetraploid maize Twf9. In next year, the pre-germinated hybrid seeds were planted in soil filled plastic pots (12 × 12 cm, inner diameter × height) and placed in experimental station of Sichuan Agricultural University for initial identification. The seedlings at 5-leaf stage were transplanted into larger plastic pots (26.5 × 26.5 cm, inner diameter × height) for further root tips collections.

### Chromosome and DNA preparation

The roots collected from parents and interspecific hybrids were immediately fixed in a saturated solution of α-bromonaphthalene for three hours, subsequently, transferred in Carnoy’s solution I (3:1 ethanol: glacial acetic acid, v/v) for 24 hours and, finally submerged in 70% ethanol solution after which these were preserved at 4 °C. Pre-mature anthers of hybrids and parents were collected and treated with Carnoy’s solution for a minimum of 12 hours and then preserved in 70% ethanol solution at 4 °C.

The preserved root tips and anthers were cleaned with distilled water to remove the effects of ethanol and then treated with an enzymatic solution comprising 6% cellulase (R-10, Yakult, Japan) and 1% pectinase (Y-23, Yakult, Japan) for 2.5–5.0 hours at 37 °C. Root tips and anthers were again thoroughly cleaned with distilled water in order to wash enzyme solution and finally, squashed onto glass slides in a drop of Carnoy’s solution I and dried with ethanol flame. The preparations showing well-spread and clean mitotic and meiotic chromosomes were selected by phase-contrast light microscopy (Olympus BX-41, Japan) and stored at -20 °C for *in situ* hybridization. Total genomic DNA from young leaves of maize and *Z. perennis* was extracted according to modified 2 × CTAB methods (Jie et al. 2015).

### Genomic *in situ* hybridization

The genomic DNA of maize and *Z. perennis* were labeled with DIG-Nick Translation and BIOTIN-Nick Translation Mix (Roche, Swiss), respectively according to manufacturer’s protocol. The selected slides were preheated in an air blowing oven at 60 °C for one hour and then transferred into 0.1ug/ml RNase (Solarbio, China) in 2 × SSC solutions in a thermostat water bath at 37 °C for one hour. Afterwards, slides were washed twice in 2 × SSC for 5 minutes each at room temperature, followed by chromosome denaturation in 70 percent deionized formamide (FAD) solution at 70 °C for 2.5 minutes, then immediately dehydrated in an ice-cold 70 percent, 95 percent and 100 ethyl alcohol series and finally air dried at room temperature. The hybridization mixture comprised 150 µl 50% FAD, 60 µl 10% dextran sulfate (DS), 30 µl 2 × SSC, 15 µl 0.5% sodium dodecyl sulfate (SDS), 30 μg salmon sperm DNA (SSDNA) and 18 µl labeled probes for six slides. Hybridization mixture was denatured in a thermostat at 85 °C for 10 minutes, followed by quick cooling in ice for 10 minutes. A total 46 μl hybridization mixture was loaded on each slide and hybridization was accomplished in an incubator at 37 °C for 20–24 hours. After hybridization slides were immersed in 20% FAD, 2 × SSC, 0.1 × SSC, respectively for 15 minutes each, at 42 °C. After that, the slides were washed in 0.1% Triton X-100 once and in 1 × PBS thrice for 5 minutes each and then air dried, at room temperature. All further steps were performed in dark, 50 µl antibody diluent, which contained anti-digoxigenin-fluorescein (0.6 µg/µl in 1 × PBS, Roche) and streptavidin-Cy-3 fluorescein (0.6% in 1 × PBS, Sigma) was applied onto air dried slides and immunodetection was done at 37 °C for one hour in an incubator. Consequently, slides were washed in 1 x PBS thrice for 5 minutes each at room temperature and air dried finally. The chromosome counterstaining was performed by 4, 6-diamidino-2-phenylindole (DAPI) solution containing 86% 1 × PBS and 14% DAPI 10ug/ml (Solarbio), and slides were observed with fluorescence microscope (Olympus BX-61, Japan) coupled with pre-fixed filter sets named as U-MNAU2 (excitation 360–370nm; emission 420–460nm and dichroic 400nm), MWIBA3 (excitation 460–495nm; emission 510–550nm and dichroic 505nm) and U-MWIG3 (excitation 530–550nm; emission 575nm IF and dichroic 570nm). The images were captured with Media Cybernetics CCD 700 (Charge Coupled Device) and Image Pro Plus 6.0 (Media Cybernetics, Inc.). Captured images were processed by Adobe Photoshop 5.1.

## Results

### Material synthesis and chromosome identification

**Figure 1.**
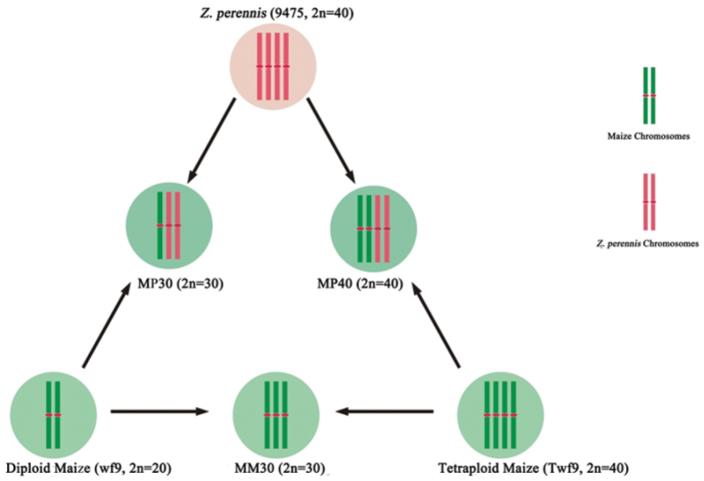
The schematic sketch of "U” triangle presents the production strategy of polyploid hybrids from one-way crosses of diploid and tetraploid parent (wf9, Twf9 and 9475) . The maize and *Z. perennis* cytoplasm are represented by light green and light pink circles, respectively. The dense green and dense pink strips represent maize and *Z. perennis* chromosomes, respectively and central red marks represents centromere of both types of chromosome

**Figure 2.**
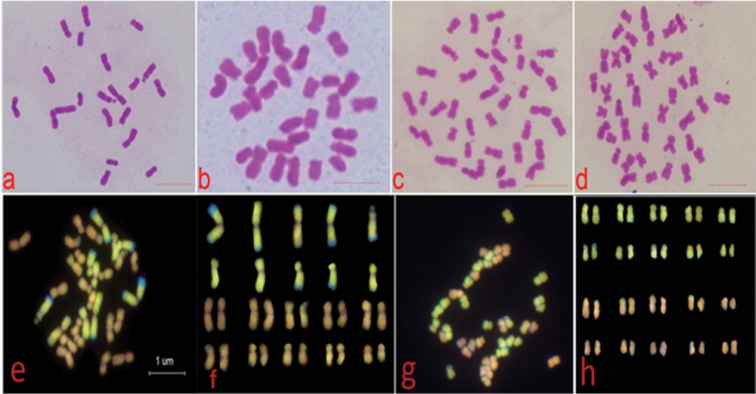
Composition of chromosomes in hybrids revealed by carbol fuchsin staining and GISH. **a, b, c, d** represents chromosome counts of wf9, MM30, Twf9, and *Z. perennis*. **e + f** and **g + h** represent chromosomal composition of MP30 and MP40, respectively. Yellow and pink signals represent maize and *Z. perennis* genome, respectively. All bars = 10 µm. The blue terminal ends of maize chromosomes represent maize knobs (intensely stained with DAPI).

Three crosses were made between diploid maize, tetraploid maize and *Z. perennis* (9475) to produce polyploid hybrids, and those synthetics are shown in Fig. 1. MP30 was an allotriploid hybrid, produced by crossing wf9 with 9475. MP40 was an allotetraploid hybrid, derived from a cross between Twf9 and 9475. MM30 was an autotriploid hybrid, produced through crossing between wf9 and Twf9. Carbol fuchsin staining was used to confirm that polyploids had been created with whole set of parental chromosomes accurately, and dual-color genomic in situ hybridization *(GISH)* was followed to authenticate the chromosome complements and composition in hybrids. The results confirmed that autotriploid maize MM30 possessed thirty maize chromosomes with 2n=3x=30, allotriploid maize MP30 (2n=3x=10) had 10 maize and 20 *Z. perennis* chromosomes, while allotetraploid maize MP40 (2n=4x=20) was consisted of 20 maize and 20 *Z. perennis* chromosomes (Fig. 2). Furthermore, we did not observe any chromosomal recombination in F_1_ hybrids. The hybrids were subjected to detailed meiotic analysis after confirming their genomic constitutions.

### Chromosome pairing in diploid maize and *Z. perennis*

**Figure 3.**
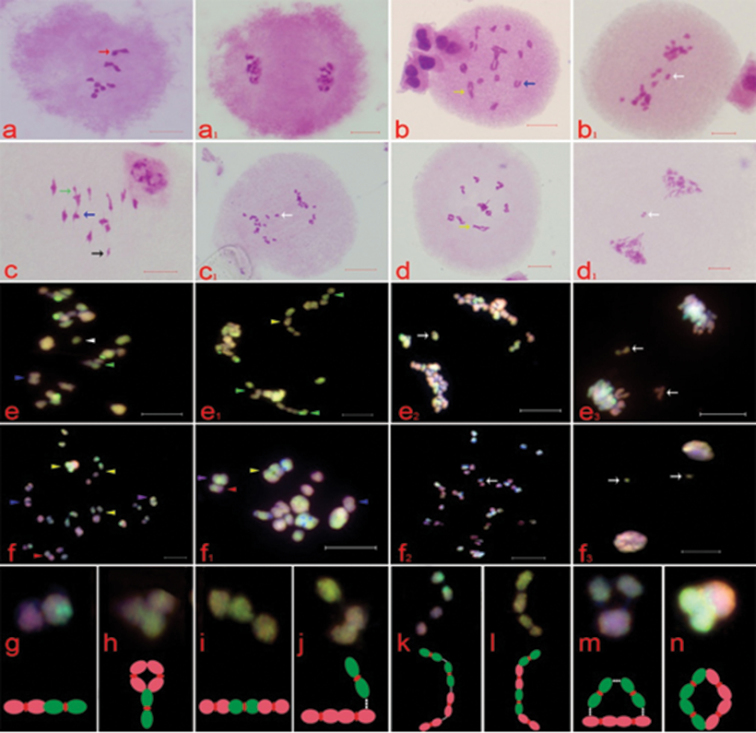
Chromosome pairing analysis of parents and hybrids. **a, b, c, d, e (e_1_), f (f_1_)** represent diakinesis of wf9, 9475, MM30, Twf9, MP30 and MP40, respectively. **a_1_, b_1_, c_1_, d_1_, e_2_, f_2_** represent meiotic anaphaseIand **e3, f3** represent meiotic telophaseI. Black arrow represents univalent, blue arrow represents bivalent, green arrow represents trivalent, yellow arrow represents quadrivalent, red arrow represents quadrivalent in diploid maize. White triangle represents univalent of maize genome. *Z. perennis* and maize autosyndetic bivalents are shown by blue and purple triangles, respectively. The allosyndetic bivalents, which were composed of one maize and one *Z. perennis* chromosome are represented by red triangles. The allosyndetic trivalents consisting of one maize and two *Z. perennis* chromosomes are represented by green triangles. Allosyndetic quadrivalents composed of two maize and two *Z. perennis* chromosomes are indicated by yellow triangle, while white arrow indicates lagging chromosomes **g, h, i, j, k, l, m, n** show different pairing types with the models below. Yellow and pink signals represent maize and *Z. perennis* genomes, respectively. All Bars = 10 µm.

Diploid maize genome exhibited regular meiosis and the most frequently observed meiotic configuration was 10II (Fig. 3a_1_; Table 4), but quadrivalents were also seen in a few PMCs, which suggested that a limited homology existed between "A” and "B” sub-genomes. The most prevalent meiotic configuration of *Z. perennis* was 10II+5IV (34.83%), and an average pairing configuration was of 0.18I+10.46II+0.13III+4.62IV (Table 4). Univalents and trivalents were rarely seen in the *Z. perennis* genome, and the prevalent numbers of bivalents and quadrivalents were ten (37.31%) and five (40.30%) with the range of 3–18 and 1–8, respectively (Fig. 3b, b_1_; Table 4). The RCC of *Z. perennis* genome was 1.13, and significantly higher than that of the maize genome (0.48), as shown in Table 1.

**Table 1.** Meiotic chromosome pairing in pollen mother cells (PMCs) of parents.

Parents	2n	I	II	III	IV	RCC	PMCs
**wf9**	20	0.01	7.31	0.01	1.33	0.48^b^	81
**9475**	40	0.18	10.46	0.13	4.62	1.13^a^	201

Note: I, II, III and IV represent univalent, bivalent, trivalent and quadrivalent, respectively. Relative chaotic coefficient (RCC) = (chromosomes number of bivalents) / (total chromosomes number - chromosomes number of bivalents). ^a, b^ Groups differed significantly by x^2^-test, p<0.05.

### Chromosome pairing in synthetic triploids

The most prevalent meiotic configuration of MM30 was 1I+4II+7III (29.67%) with the average of 0.71I+3.31II+7.19III+0.28IV, while 10III (11.72%) was also found in some PMCs (Table 4), and the lagging chromosomes were frequently observed at first anaphase stage (Fig. 3c_1_). Nearly half of analyzed PMCs did not show univalents and the average (range) number was 0.71 (0–3). The most frequent number of bivalents was four (24.00%) with an average (range) number of 3.31 (0–9) that suggests some of the paired chromosomes in maize genome didn’t share complete homology. MM30 had abundant trivalent, the most repeated number was eight (24.00%) and an average (range) number was 7.17 (2–10). On the contrary with wf9, most of PMCs in MM30 did not contain quadrivalents and an average (range) number was 0.28 (0–3), as shown in Tables 2, 4. The MM30 showed irregular meiotic behavior and its RCC was 4.87, higher than that of wf9 (Table 2).

**Table 2.** Average number of meiotic chromosomes associations in PMCs of triploid hybrids verified by GISH.

**Hybrids**	**2n**	**I**			**II**				**III**			**IV**	**RCC**			**PMCs**
		**Total**	**I^M^**	**I^P^**	**Total**	**II^MM^**	**II^PP^**	**II^MP^**	**Total**	**III^MPP^**	**Others**	**Total**	**Total**	**wf9**	**9475**	
**MM30**	30	0.71^b^	0.71^b^	–	3.31^b^	3.31^a^	–	–	7.19^a^	–		0.28^a^	4.78^a^	4.78^b^	–	129
**MP30**	30	4.56^a^	3.79^a^	0.77	5.44^a^	0.25^b^	4.34	0.85	4.76^b^	4.55	0.21	0.07^b^	2.59^b^	9.00^a^	1.56	71

Note: I, II, III and IV represent univalent, bivalents, trivalents and quadrivalents. I^M^ and I^P^ represent maize and *Z. perennis* univalent, respectively. II^MM^ and II^PP^ represent autosyndetic bivalents of maize and *Z. perennis*, respectively. II^MP^ represents allosyndetic bivalents having one chromosome from maize genome and one chromosome from *Z. perennis*. III^MPP^ represents allosyndetic trivalents, which were composed of one chromosome from maize genome and two chromosomes from *Z. perennis* genome. ^a, b^ Groups differed significantly by x^2^-test, p<0.05.

The PMCs of MP30 frequently showed lagging chromosomes at meiotic anaphase I (Fig. 3e_2_, e_3_). Dual-color genomic in situ hybridization was carried out to study cryptic chromosome pairing in synthesized allopolyploid (Fig. 3). An average pairing configuration was 4.56I+5.44II+4.73III+0.07IV (Table 4), and the most common meiotic pairing configuration was 5III+5II+5I (16.9%).Based on the result of GISH, most univalents in MP30 were from maize (I^M^), and most common number was five (18.30%) with an average number (range) of 3.79 (0–8). There were also some univalents from *Z. perennis* (I^P^) with most repeated number of zero (57.75%) and an average number (range) was 0.77 (0–7). Most autosyndetic bivalents in MP30 were from *Z. perennis* (II^PP^) with the most common number of five (26.76%) and an average number (range) of 4.34 (1–7). The other autosyndetic bivalents were from maize genome (II^MM^) with an average number of 0.25 (0–1), whereas allosyndetic bivalents (II^MP^) were rarely seen in MP30, as their average numbers (range) was 0.85 (0–4). Most of the trivalents were allosyndetic trivalents (III^MPP^), which were composed of one chromosome from maize and two chromosomes from *Z. perennis*, and most the frequent number was five (36.62%) with an average number of 4.55 (0–7) as shown in Table 2. These results suggest that five chromosomes from maize genome in MP30 were homologous to ten chromosomes of *Z. perennis*. Moreover, quadrivalents and autosyndetic trivalents were rarely seen in MP30 (Table 4).

For more detailed analysis of chromosomes in MP30, the configuration of III^MPP^ was examined. The configuration of III^MPP^ was not similar in all cases (Fig. 3h, i, j and Table 5). The most frequent configuration of III^MPP^ was "frying pan type” with an average number (range) of 3.23 (0–6), Fig. 3h. However, there was also another configuration that was "rod type” with an average number (range) of 1.18 (0–5), suggesting that some paired chromosomes in "A” subgenome of *Z. perennis* were discrepant homologous (Fig. 3i, j and Table 5). Additionally, there were also some allosyndetic trivalents in which maize chromosomes were associated with *Z. perennis* chromosome loosely, which suggested that evolutionary differentiation had occurred in the "A" subgenome of maize and *Z. perennis* (Fig. 3j). Comparative analysis of MM30 and MP30 showed that the RCC of maize genome in MM30 (4.78) was lower than in MP30 (9.00) and autosyndetic bivalents of maize genome in MP30 were much lower than MM30. The *Z. perennis* chromosomes in MP30 had lower RCC than that of maize in MM30 and MP30. Thus, overall RCC of MP30 was lower than that of MM30.

### Chromosome pairing in synthetic tetraploids

The most frequent meiotic configuration of MM40 was 10IV (21.67%) with the average of 0.26I+3.61II+0.14III+8.03IV (Table 4), and the lagging chromosomes in PMCs were found commonly at meiotic anaphase I. More than half of PMCs did not show univalents and trivalents with average number (ranges) for univalents and trivalents being 0.26 (0–2) and 0.14 (0–2), respectively. Moreover, commonest number of bivalents was zero (25.00%) with an average number (range) of 3.61 (0–10). MM40 had abundant quadrivalents and the most frequent number was nine (24.17%) with an average (range) number of 8.03 (4–10), as shown in Tables 3, 4. These results also suggest that some paired chromosomes in maize didn’t share complete homology. The MM40 showed anomalous meiosis and its RCC was 4.83, higher than wf9 (Table 3).

**Table 3.** Average number of chromosomes associations in PMCs of tetraploid hybrids revealed by GISH.

Hybrids	2n	I			II				III	IV			RCC			PMCs
		Total	I^M^	I^P^	Total	II^MM^	II^PP^	II^MP^	Total	Total	IV^MMPP^	Others	Total	wf9	9475	
**MM40**	40	0.26^b^	0.26^b^	–	3.61^b^	3.61^b^	–	–	0.14^a^	8.03^a^	–	–	4.83^a^	4.83^a^	–	121
**MP40**	40	1.17^a^	0.81^a^	0.36	9.97^a^	4.30^a^	4.71	0.96	0.13^a^	4.62^b^	4.29	0.33	1.46^b^	1.60^b^	1.47	69

Note: The I, II, III and IV symbolize univalent, bivalent, trivalent and quadrivalent, and I^M^ and I^P^ represent maize and *Z. perennis* univalents, respectively. II^MM^ and II^PP^ represent bivalent composed of two chromosomes of maize and two chromosomes of *Z. perennis*, respectively. II^MP^ represents allosyndetic bivalent consists of one chromosome from maize and one chromosome from *Z. perennis*. IV^MMPP^ represents allosyndetic quadrivalents composed of two chromosomes from maize and two chromosomes from *Z. perennis*. ^a, b^ groups differed significantly by x^2^-test, p<0.05.

The most common meiotic configurations of allotetraploid maize (MP40) were 8II+6IV (15.94%) and 12II+4IV (15.94%), and the lagging chromosomes found at meiotic anaphase I (Fig. 3f2, f3). However, a rare meiotic configuration 10II+5IV (13.04%) was also observed, with an average number of 1.17I+9.97II+0.13III+4.62IV. More than half of PMCs didn’t show univalents, and average (range) number of maize and *Z. perennis* genome’s univalents were 0.81 (0–8) and 0.36 (0–7), respectively. The autosyndetic bivalents from maize and *Z. perennis* were frequently appeared and the most prevalent number was five (24.64%; 27.54%) for both, while average number (ranges) for maize and *Z. perennis* were 4.30 (2–7) and 4.71 (1–9), respectively. Most PMCs did not possess allosyndetic bivalents and the average number (range) was 0.96 (0–4). The trivalents existed in several PMCs; in addition, maize autosyndetic trivalents were not found. Most PMCs did not contain autosyndetic quadrivalents and most allosyndetic quadrivalents (IV^MMPP)^ consisted of two chromosomes from *Z. perennis* and two chromosome from maize, with most prevalent number of five (24.64%) and an average number was 4.29 (1–7) as shown in Tables 3, 4. These results suggest that ten chromosomes from maize genome in allotetraploid were homologous to ten chromosomes of *Z. perennis* genome.

The detailed chromosome observation of MP40 showed that configurations of IV^MMPP^ were not similar. The most frequent configuration of IV^MMPP^ was of "ring type” with an average number (range) of 0.72 (0–6), while another form of "rod type” also found (Fig. 3k, l, m, n and Table 5). In addition, some allosyndetic quadrivalents were also seen in which maize chromosome were weekly associated with *Z. perennis* chromosomes (Fig. 3k, m). The different configurations of IV^MMPP^ suggested that some paired chromosomes of "B” subgenome of maize and *Z. perennis* were discrepantly homologous. Similarly, the "B” subgenome has undergone considerable evolutionary differentiation in the genus *Zea* but the "A” subgenome has undergone only slight differentiation.

The comparative analysis of MM40 and MP40 revealed that the RCC of maize genome in MM40 was higher than MP40, suggested that a limited homology between maize and *Z. perennis* genomes enhance meiotic stability in maize allotetraploid. Comparative analysis between *Z. perennis* and MP40 showed higher number of bivalents and lower RCC in *Z. perennis* than MP40 and Twf9 that might be due to allopolyploid nature of *Z. perennis* (Naranjo et al. 1994).

## Discussion

### Dissimilar meiotic stabilities between maize autopolyploids and allopolyploids

Polyploidy is a state in which more than two sets of chromosomes coexist in one nucleus. It is a widespread phenomenon in plants and is considered to be a major force in plant evolution (Comai 2000, Lavania 2013). The autopolyploids have three or more homologous chromosomes and can form multivalents during meiosis so that meiotic stability is a bottleneck for their sexual reproduction (Soltis and Soltis 2000). In allopolyploids, homologous genome causes autosyndesis, while different genome in one nucleus can hardly induce allosyndesis as well. The diploid paring model is strictly enforced in allopolyploids in which parental genomes have limited affinity (Wu et al. 2001). However, to the best of our knowledge, there are also some allopolyploids that possess homologous or homoeologous chromosomes between parental genomes, thus they do not follow diploid paring model strictly. They form univalents and/or multivalents that cause meiotic confusion and genetic instability (Eckardt 2001). Furthermore, meiosis of autopolyploids is generally less stable than allopolyploids. In our study, we also found the consistent observations with those that have been previously reported. The RCC of MP30 and MP40 was lower than MM30 and MM40, respectively that might be as a result of discrepant homology that exists between maize and *Z. perennis* chromosomes (Wang et al. 2011). The number of autosyndetic bivalents in allotetraploid maize was higher than autotetraploid maize. On the contrary, the RCC of maize allopolyploids was higher than diploid maize. Perhaps the reason for higher RCC of allopolyploids is occurrence of homoeologous chromosomes between maize and *Z. perennis* genome, so that allosyndesis and multivalency can be expected (González et al. 2006).

### Genetic relationship between maize and *Z. perennis*

The maize genome has a large number of duplicated genes according to theory of tetraploid origin (Ahn et al. 1993, Wendel 2000, Gaut et al. 2000, Doerks et al. 2002, Wang et al. 2011) (Doerks et al. 2002; Molina et al. 2012). For diploid maize and diploid teosinte hybrids (2n=20), the two groups of five bivalents were observed at meiosis, which suggested that genome can be divided into "AA” and "BB” sections (Naranjo et al. 1994). Quadrivalents were observed in diploid maize (Tables 1, 4) and the same phenomenon was also reported previously (Molina et al. 2013). These results suggested that "A” and "B” subgenomes are homoeologous in maize. The earliest suggested genomic formula for Z. mays ssp. mays was A_2_A_2_ B_2_B_2_ and for *Z. perennis* is A_l_’A_l_’A_1_”A_1_” C_l_C_l_ C_2_C_2_. Additionally, homoeologous genomes usually do not pair, maybe due to the presence of *Ph*-like gene (Poggio et al. 1990). Hexavalent were not seen in triploid maize, as well as octavalent were also not observed in tetraploid maize (Table 4), which suggested a limited paring between "A” and "B” subgenomes at higher ploidy levels. In addition, the most frequent number (range) of autosyndetic trivalent in PMCs of MM30 was eight (2–10) and common number (range) of autosyndetic quadrivalent in MM40 was nine (4–10), that indicated some paired chromosomes in "A” subgenome or in "B” subgenome have been differentiated. Furthermore, the *Z. perennis* belongs to another section of genus *Zea* (Iltis and Doebley 1980) and has a hypothetical octoploid origin that was also confirmed by genetic linkage maps (Moore et al. 1995). The maximum number of bivalents and quadrivalents in PMCs were 18 and 8, respectively suggesting that "A” subgenome have been subjected to evolutionary differentiation but homologous relationship still exists in "B” subgenomes. Hexavalent and octavalent were not seen in *Z. perennis*, as well as in colchicine treated doubled diploid maize. However colchicine treatment could initiate paring of "B” subgenome with a maximum number of 10IV. These results revealed that homoeologous relationship exists in "B” subgenome of *Z. perennis* (Molina et al. 2013).

**Table 4.** Meiotic chromosome pairings in PMCs of parents and hybrids.

Materials			wf9		9475		MM30		MP30		MM40		MP40		
Average configuration			0.01I+7.31II +0.01III+1.33IV		0.18I+10.46II +0.13III+4.62IV		0.71I +3.31II +7.19III+0.28IV		4.56I+5.44II +4.73III+0.07IV		0.26I+3.61II +0.14III+8.03IV			1.17I+9.97II +0.13III+4.62IV	
Frequent configurations			10II (35.00)		10II+5IV (34.83)		1I+4II+7III (29.67)	10III (11.72)	5I+5II+5III (16.9)		10IV (21.67)		8II+6IV (15.94)	12II+4IV (15.94)	10II+5IV (13.04)
Frequent valents (Range)	I (%)	Total	0 (98.77)		0 (88.56)		0 (51.9) –		5 (25.3)		0 (80.83)		0 (52.1)		
		I^M^	0 (98.77)	(0–1)			0 (51.9)	(0–3)	5 (18.3)	(0–8)	0 (80.83)	(0–2)	0(55.07)	(0–5)	
		I^P^	–	–	0 (88.56) –	(0–4)–	–		0(57.75)	(0–7)	–	–	0(71.01)	(0–2)	
	II (%)	Total	8 (33.33)		10 (37.3)		4 (24.0)		5 (29.58)		0 (25.00)		10 (17.3)		
		II^MM^	8 (33.33)	(0–10)			4 (24.0)	(0–9)	0(74.65)	(0–1)	0 (25.00)	(0–10)	5(24.64)	(2–7)	
		II^PP^	–	–	10 (37.31) –	15	–		5(26.76)	(1–7)	–	–	5(27.54)	(1–9)	
		II^MP^	–	–	–	–	–		0(52.11)	(0–4)	–	–	0(59.42)	(0–4)	
	III (%)	Total	0 (98.77)		0 (90.55)		8 (24.0)		5 (33.8)		0 (86.67)		0(88.41)		
		III^MMM^	0 (98.77)	(0–1)			8 (24.0)	(2–10)	0 (95.2)	(0–1)	0 (86.67)	(0–2)	0 (100)		
		III^PPP^	–	–	0 (90.55)	(0–3) –	–		0(90.14)	(0–1)	–	–	0(94.20)	(0–1)	
		III^MMP^	–	–	–	–	–		0(91.55)	(0–3)	–	–	0(97.10)	(0–1)	
		III^MPP^	–	–	–	–	–		5(36.62)	(0–7)	–	–	0(94.20)	(0–1)	
	IV (%)	Total	1 (34.57)		5 (40.30)		0 (77.5)		0 (92.96)		9 (24.17)		5 (26.09)		
		IV^MMMM^	1 (34.57)	(0–5)			0 (77.5)	(0–3)	0 (100)		9 (24.17)	(4–10)	0(81.16)	(0–3)	
		IV^PPPP^	–	–	5 (40.30) –	(1–8)	–		0(97.18)	(0–1)	–	–	0(89.86)	(0–1)	
		IV^MMPP^	–	–	–	–	–		0(95.77)	(0–1)	–	–	5(24.64)	(1–7)	

Chromosome pairings between maize and *Z. perennis* was observed in PMCs of two allopolyploids. We found univalents, bivalents and multivalents and allosyndetic valents at different levels during meiosis. The meiotic configuration of MP30 was 5I^M^+5II^PP^+5III^MPP^, while univalents I^M^, bivalents II^PP^ and allosyndetic trivalents III^MPP^ were common. The meiotic configuration of MP40 was 5II^MM^ +5II^PP^ +5IV, while bivalents II^MM^, bivalents II^PP^ and allosyndetic quadrivalents IV^MMPP^ were frequently observed (Table 4), which reveals that genetic relationship exists in maize and *Z. perennis*. Additionally, ten chromosomes of "A” subgenome in maize are homologous with twenty chromosomes of "A” subgenome in *Z. perennis*, on the contrary, "B” subgenome has been highly differentiated (Fukunaga et al. 2005; Swanson-Wagner et al. 2010). Comparatively, the levels and frequency of auto- and allosyndesis for each genome as well as meiotic configuration were not in well agreement with previous findings (Naranjo et al. 1994, Poggio et al. 1999, González et al. 2006, Molina et al. 2013). The possible explanations include: (a) Different maize cultivars were used and genomes of those maize cultivars might be slightly different; (b) Different circumstance and different maize cultivar, as well as different genome composition in polyploids might influence the expression of *Phs1* and *Pam1* genes that play an important role in homologous chromosomes pairing (Golubovskaya et al. 2002, Ronceret et al. 2009, Lukaszewski and Kopecký 2010, Feddermann et al. 2010, Ianiri et al. 2014). In addition, autosyndetic bivalents of the maize genome were rarely seen in MP30 and maximum number of III^MPP^ in MPCs was seven. It suggested that homology of ten maize chromosomes in MP30 was extremely low and homologous relationship exists in "B” subgenome, as well as homoeologous relationship existed in "A” subgenome of maize and *Z. perennis*. The *Z. perennis* chromosomes in MP30 had lower RCC than RCC of maize chromosomes in MM30 and MP30, thus, overall RCC of MP30 was lower than MM30, which suggested the limited homology between maize and *Z. perennis* enhance overall meiotic stability in maize allotriploid. In MP40, the maximum number of autosyndetic bivalents, which belong to maize and *Z. perennis* genome, was seven and nine, respectively; otherwise, the maximum number of IV^MMPP^ was also seven. The minimum number of IV^MMPP^ was one, which suggested that "A” subgenome between maize and *Z. perennis* shared partial homology, and maximum number of IV^MMPP^ seven suggested that limited homologous relationship existed in "B” subgenome of maize and *Z. perennis*.

**Figure 4.**
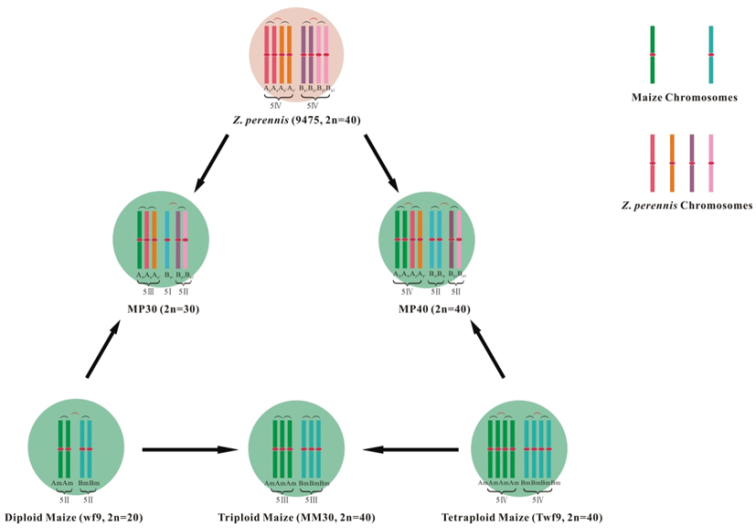
Schematic genomic diagram of maize, *Z. perennis* and hybrids.

Detailed examination of allosyndetic trivalents (III^MPP^) revealed that there were not only "frying pan type”, but "rod type” also existed in allotriploid with a maximum number of five (Table 5). These results are consistent to previous study (González et al. 2006). In addition, prevalent configuration of allosyndetic quadrivalents (IV^MMPP^) was not only the "rod type” but "ring type” was also found (Table 5). In III^MPP^ and IV^MMPP^, the degree of chromosome pairing was variable e.g. relatively tight chromosome pairing between maize and *Z. perennis*, maize and maize, *Z. perennis* and *Z. perennis* were observed, while the loose chromosome pairing between maize and *Z. perennis*, maize and maize, *Z. perennis* and *Z. perennis* was also seen (Figure 3j, k, m). These results suggested that "A” subgenomes in two parents underwent evolutionary differentiation but at lower degree as compared to "B” subgenomes. The schematic genomic formula representation of maize, *Z. perennis* and their hybrids is built (Fig. 4).

**Table 5.** Types of allosyndetic trivalents and quadrivalents.

**Valente types**		**MP30**		**MP40**	
IIIfry-pan type	Mean (Range)	3.23 (0–6)		–	
	Frequency (%)	3 (32.39)		–	
		4 (18.31)		–	
		5 (22.54)		–	
IIIrod type	Mean (Range)	1.18 (0–5)		–	
	Frequency (%)	0 (33.80)		–	
		1 (30.99)		–	
		2 (23.94)		–	
IVring type	Mean (Range)	–		2.78 (0–6)	
	Frequency (%)	–		2 (26.76)	
		–		3 (26.76)	
		–		1,4,5 (14.08)	
IVrod type	Mean (Range)	–		0.72 (0–6)	
	Frequency (%)	–		0 (45.07)	
		–		1 (38.03)	
		–		2 (11.27)	

**Table 6.** Plant material used in the study.

Scientific name	Source	Accession	Chromosome number
*Zea perennis*	CIMMYT	9475	2n = 40
Zea mays ssp. mays	USDA	wf9	2n = 20
Zea mays ssp. mays	USDA	Twf9	2n = 40

The maize and *Z. perennis* cytoplasm are represented by light green and light pink circles, respectively. The blue and green strips represent maize chromosomes, while pink, orange, brown and dark red strips represent *Z. perennis* chromosomes. The centromeres in middle of all chromosomes are labeled red; moreover, both of them have red centromere in the middle. Black parentheses represent paired homologous chromosomes and red parentheses represent expected chromosomes combinations.

### Expected evolutionary mechanism of maize and *Z. perennis*

**Figure 5.**
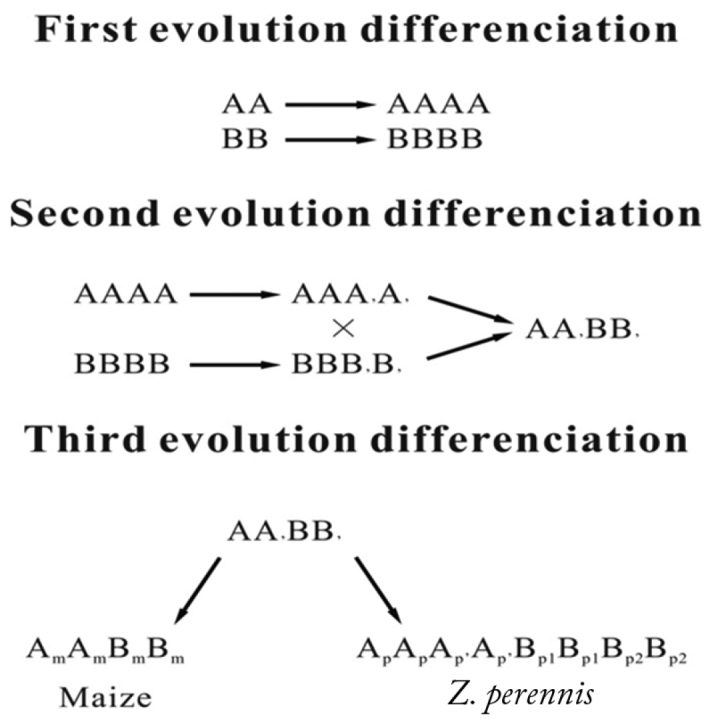
Possible mechanisms of genome differentiation in maize and *Z. perennis*.

In previous studies, two possible evolutionary mechanisms for maize and *Z. perennis* genome were proposed: First, the genome composition of "AABB” hybrids was an ancestral cross between two closely related "AA” and "BB” genomes that was followed by evolutionary fractionation; Second, *Zea* species were originated through chromosome duplication, followed by homoeologous genomes "A” and "B” differentiation (Molina et al. 2013). However, both hypotheses cannot explain differences within "A” and "B” subgenomes in both maize and *Z. perennis* appropriately. Thus, we put forward a third possible evolutionary mechanism: Firstly, duplication event occurred in two closely related species with "AA” and "BB” genome, as a consequence, autopolyploid of "AAAA” and "BBBB” genome were formed. Secondly, evolutionary fractionation took place in two autopolyploids that turned both genomes into "AAA_’_A_’_” and "BBB_’_B_’_”. thirdly, crossing between those two autopolyploids followed by probable limited compatible coevolution in "A” and "B” subgenomes led to the formation of "A_m_A_m_B_m_B_m_” genome with barely deviation of maize intra-subgenomes; Lastly, second duplication event of hybrids "AABB_’_” followed by differential degree of evolutionary fractionation in "A” and "B” subgenomes, led to creation of *Z. perennis* with genome of "A_p_A_p_A_p’_A_p’_B_p1_B_p1_B_p2_B_p2_” (Fig. 5). Moreover, as "A” genome has higher homology between maize and *Z. perennis* than "B” genome and also suffers less genes losses, as well as, has higher expression for genes located in this subgenome, while "B” genome has a faster differentiation. So it is concluded that "B” subgenome was responsible for species isolation, domestication, and as well as further speciation in genus *Zea* (Freeling and Thomas 2006, Swanson-Wagner et al. 2010, Schnable et al. 2011).

## Supplementary materials

Supplementary material 1 Supplementary tables. This is an open access article distributed under the terms of the Creative Commons Attribution License (CC BY 4.0), which permits unrestricted use, distribution, and reproduction in any medium, provided the original author and source are credited.Muhammad Zafar Iqbal Mingjun Cheng, Yanli Zhao, Xiaodong Wen, Ping Zhang, Lei Zhang, Asif Ali, Tingzhao Rong, Qi Lin Tang
