# Supplementary material for: Mysterious meiotic behavior of autopolyploid and allopolyploid maize
Source: Comp Cytogenet. 2018 Jul 20;12(2):247–65. doi: 10.3897/CompCytogen.v12i2.24907 (PMC6063980; doi:10.3897/CompCytogen.v12i2.24907)
Supplement: Supplementary material 1 — Supplementary tables. [file comparative_cytogenetics-12-247-s001.docx]

Table 1S Analysis meiosis chromosome pairings of parents and hybrids

| Materials | Average Configuration | Frequent Configurations | Frequent Valents (Range) | | | | | | | | | | | | | | | |
| --- | --- | --- | --- | --- | --- | --- | --- | --- | --- | --- | --- | --- | --- | --- | --- | --- | --- | --- |
|  |  |  | A (%) | | | B (%) | | | | C (%) | | | | | D (%) | | | |
|  |  |  | Total | Ⅰ^M^ | Ⅰ^P^ | Total | Ⅱ^MM^ | Ⅱ^PP^ | Ⅱ^MP^ | Total | Ⅲ^MMM^ | Ⅲ^PPP^ | Ⅲ^MMP^ | Ⅲ^MPP^ | Total | Ⅳ^MMMM^ | Ⅳ^PPPP^ | Ⅳ^MMPP^ |
| wf9 | 0.01Ⅰ+7.31Ⅱ+0.01Ⅲ+1.33Ⅳ | 10Ⅱ (35.00) | 0 (98.77) | 0 (98.77) | - | 8 (33.33) | 8 (33.33) | - | - | 0 (98.77) | 0 (98.77) | - | - | - | 1 (34.57) | 1 (34.57) | - | - |
|  |  |  |  | (0-1) | - |  | (0-10) | - | - |  | (0-1) | - | - | - |  | (0-5) | - | - |
| 9475 | 0.18Ⅰ+10.46Ⅱ+0.13Ⅲ+4.62Ⅳ | 10Ⅱ+5Ⅳ (34.83) | 0 (88.56) |  | 0 (88.56)- | 10 (37.3) |  | 10 (37.31)- | - | 0 (90.55) |  | 0 (90.55) | - | - | 5 (40.30) |  | 5 (40.30)- | - |
|  |  |  |  |  | (0-4)- |  |  | - (3-18) | - |  |  | (0-3)- | - | - |  |  | (1-8) | - |
| MM30 | 0.71I +3.31II +7.19Ⅲ+0.28IV | 1Ⅰ+4Ⅱ+7Ⅲ (29.67) | 0 (51.9)- | 0 (51.9) | - | 4 (24.0) | 4 (24.0) | - | - | 8 (24.0) | 8 (24.0) | - | - | - | 0 (77.5) | 0 (77.5) | - | - |
|  |  | 10Ⅲ (11.72) |  | (0-3) |  |  | (0-9) |  |  |  | (2-10) |  |  |  |  | (0-3) |  |  |
| MP30 | 4.56I+5.44II+4.73III+0.07IV | 5Ⅰ+5Ⅱ+5Ⅲ (16.9) | 5 (25.3) | 5 (18.3) | 0(57.75) | 5 (29.58) | 0(74.65) | 5(26.76) | 0(52.11） | 5 (33.8) | 0 (95.2) | 0(90.14) | 0(91.55) | 5(36.62) | 0 (92.96) | 0 (100) | 0(97.18) | 0(95.77) |
|  |  |  |  | (0-8) | (0-7) |  | (0-1) | (1-7) | (0-4) |  | (0-1) | (0-1) | (0-3) | (0-7) |  |  | (0-1) | (0-1) |
| MM40 | 0.26Ⅰ+3.61Ⅱ+0.14Ⅲ+8.03Ⅳ | 10Ⅳ (21.67) | 0 (80.83) | 0 (80.83) | - | 0 (25.00) | 0 (25.00) | - | - | 0 (86.67) | 0 (86.67) | - | - | - | 9 (24.17) | 9 (24.17) | - | - |
|  |  |  |  | (0-2) | - |  | (0-10) | - | - |  | (0-2) | - | - | - |  | (4-10) | - | - |
| MP40 | 1.17I+9.97II+0.13III+4.62IV | 8Ⅱ+6Ⅳ (15.94) | 0 (52.1) | 0(55.07) | 0(71.01) | 10 (17.3) | 5(24.64) | 5(27.54) | 0(59.42) | 0(88.41) | 0 (100) | 0(94.20) | 0(97.10) | 0(94.20) | 5 (26.09) | 0(81.16) | 0(89.86) | 5(24.64) |
|  |  | 12Ⅱ+4Ⅳ (15.94) |  | (0-5) | (0-2) |  | (2-7) | (1-9) | (0-4) |  |  | (0-1) | (0-1) | (0-1) |  | (0-3) | (0-1) | (1-7) |
|  |  | 10Ⅱ+5Ⅳ (13.04) |  |  |  |  |  |  |  |  |  |  |  |  |  |  |  |  |

Table 2S the types of allosyndetic trivalents and allosyndetic quadrivalents

| Valente Types | Ⅲfry-pan type | | | | Ⅲrod type | | | | Ⅳring type | | | | Ⅳrod type | | | |
| --- | --- | --- | --- | --- | --- | --- | --- | --- | --- | --- | --- | --- | --- | --- | --- | --- |
|  | Mean (Range) | Frequent (%) | | | Mean (Range) | Frequent (%) | | | Mean (Range) | Frequent (%) | | | Mean (Range) | Frequent (%) | | |
| MP30 | 3.23 (0-6) | 3 (32.39) | 4 (18.31) | 5 (22.54) | 1.18 (0-5) | 0 (33.80) | 1 (30.99) | 2 (23.94) | - | - | - | - | - | - | - | - |
| MP40 | - | - | - | - | - | - | - | - | 2.78 (0-6) | 2 (26.76) | 3 (26.76) | 1,4,5 (14.08) | 0.72 (0-6) | 0 (45.07) | 1 (38.03) | 2 (11.27) |
